# Enhancing the antitumor potency of T cells redirected by bispecific antibodies

**DOI:** 10.18632/oncoscience.366

**Published:** 2017-10-05

**Authors:** Chien-Hsing Chang, David M. Goldenberg

**Affiliations:** IBC Pharmaceuticals Inc., and Immunomedics Inc., Morris Plains, New Jersey, USA

**Keywords:** immunotherapy, T cells, bispecific antibody, CD3, Trop-2

**Figure 1:**
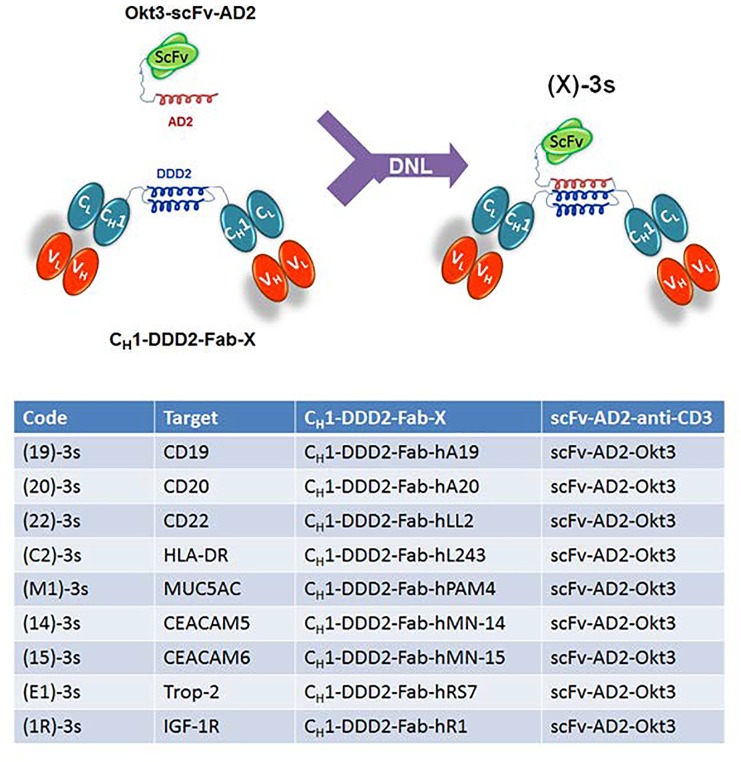
A list of (X)-3s made by the DNL® with a schematic showing the site-specific conjugation between a dimer of anti-X Fab and a monomer of anti-CD3 scFv via the fused DDD2 and AD2 peptides

The idea that a bispecific antibody might activate and simultaneously engage T cells to kill targeted tumor cells was conceived more than 30 years ago (1). Since then, two T-cell-redirecting bispecific antibodies (bsAbs) of different designs, catumaxomab and blinatumomab, have been approved by regulatory agencies, with many others at various stages of preclinical and clinical development (2). To date, the ongoing efforts to optimize the therapeutic potential of bispecific antibodies in general, and those intended to redirect immune cells, including T cells, NK cells, and Treg cells, in particular, have led to a plethora of functional constructs distinguishable by diverse formats and different specificities (3). During the past decade, our group has devised the Dock-and-Lock® (DNL®) platform that combines recombinant engineering and site-specific conjugation to create multispecific, multivalent antibodies of defined composition with retained bioactivity (4). For example, we have applied DNL® to generate a novel class of trivalent bsAbs, each comprising an anti-CD3 scFv covalently conjugated to a stabilized dimer of different anti-tumor Fabs. These DNL® conjugates were designated (X)-3s, where the codes (X) and 3s denote the bivalent anti-tumor Fab dimer and the monovalent anti- CD3 scFv, respectively, as depicted in Fig. 1. In our first publication (5), they were shown to mediate the formation of immunological synapses between T cells and cognate target cells, induce T-cell activation and proliferation in the presence of target cells, kill target cells at subnanomolar IC_50_ 's when co-cultured with T cells *in vitro*, and inhibit growth of human tumor xenografts in NOD/SCID mice reconstituted with human PBMCs. We also demonstrated in a follow-on study with (E1)-3s, a representative (X)- 3s with specificity for Trop-2-expressing epithelial cancer cells, that the addition of interferon-α (IFN-α) further enhances the potency of redirected T cells *in vitro* without a significant increase in cytokine production, and that the combination of (E1)-3s with peginterferon alfa-2a more effectively delayed the growth of Trop-2-expressing NCI-N87 human gastric cancer xenografts *in vivo* than single treatments with either (E1)-3s or peginterferon alfa-2a, alone or when combined (6). Additionally, we noted the CD19-targeting (19)-3s induced considerably lower amounts of INF-γ, TNF-α, IL-2, IL-6, and IL- 10, when compared with the 19-3 BiTE counterpart made as a biosimilar to blinatumomab. Because the inevitable discharge of inflammatory cytokines, such as TNF-α, IFN-γ, and IL-6, by activated T cells is a known complication commonly occurring in patients soon after the administration of T-cell-engaging therapeutics, and can be fatal in the severe case of cytokine storm (7), the relatively low levels of cytokine release triggered by (X)-3s could be a potential advantage over other types of T-cell-redirected bsAbs, including, BiTE constructs.

In a more recent study (8), we further explored the potential utility of (E1)-3s for treating Trop-2-expressing breast cancers, including triple-negative breast cancer (TNBC), as well as that of (14)-3s for CEACAM5- expressing colonic cancers. Both (E1)-3s and (14)-3s were highly potent for their target tumor cells, exhibiting largely subnanomolar IC_50c_'s against diverse cell lines grown in monolayer cultures, with positive results also discernable semi-quantitatively for (E1)-3s and (14)-3s in 3D spheroids, which have been widely recognized to better reflect the physiology and microenvironment of tumor cells *in vivo*, and are being used increasingly in cancer research to complement monolayer cultures for drug testing. More importantly, we have provided supporting evidence with monolayer cultures, 3D spheroids, and *in vivo* xenograft models that the addition of chimeric anti-PD-1, a proprietary checkpoint inhibitor, could significantly enhance (E1)-3s-mediated T-cell killing of MDA-MB-231 TNBC cells, which constitutively express PD-L1.

Whereas the cytolytic efficiency of (14)-3s, (E1)-3s, and other T-cell-redirecting bsAbs obviously requires the presence of cognate antigens on target cells, their ultimate potency in a certain cancer may depend on additional factors, such as the mutational status, epitope specificity and density on the cell surface, and the expression of intrinsic and induced immune evasion molecules that result from the stimulatory and inhibitory interplays induced by such bsAbs upon linking effector T cells to target tumor cells. Of note, accumulating data from us and others have now indicated the ligation of CD3 on effector T cells with the respective cell-surface antigen on tumor cells by a T-cell redirecting bsAb triggers the activation of T cells, which release perforin and granzyme B to kill target tumor cells, secrete IFN-γ to induce the expression of PD-L1 and IDO (indoleamine 2,3-dioxygenase) on target tumor cells, and upregulate PD-1. Moreover, blockade of PD-1/PD-L1 and other immune checkpoint inhibitory pathways may augment the antitumor immunity of T cells to reverse the ensuing T cell exhaustion and tumor evasion, yet can also enhance A2A adenosine receptor expression on T cells, leading to inhibition of T cells by adenosine produced from CD73-expressing tumor cells. Collectively, it has become clear that the antitumor immunity of T cells induced upon ligation with a bispecific antibody to target tumor cells also incurs a concurrent activation of various cell-bound as well as -secreted factors to promote tumor growth. Thus, understanding the relative contribution of each of these processes in the destruction and protection of tumor should lead to a more effective combination immunotherapy of cancer with T-cell-redirecting bsAbs.
